# Cancer Cachexia Prevalence Is Underestimated in Medical Records of Patients in a Regional Tertiary Hospital

**DOI:** 10.1002/jcsm.70225

**Published:** 2026-02-18

**Authors:** Yann Colardelle, Melanie Daligault, Julien Grosjean, Stefan J. Darmoni, Badisse Dahamna, Richard B. Weiskopf, Vickie E. Baracos

**Affiliations:** ^1^ Society on Sarcopenia, Cachexia and Wasting Disorders (SCWD) Duluth Georgia USA; ^2^ Univ Rouen Normandie, Université de Caen Normandie, INSERM, Normandie Univ, DYNAMICURE UMR 1311, CHU Rouen Rouen France; ^3^ Univ Rouen Normandie, RecSan, AIMS Lab, Digital Health Department, CHU Rouen Rouen France; ^4^ University of California San Francisco California USA; ^5^ University of Alberta Edmonton Canada

**Keywords:** cachexia, cancer, diagnosis, ICD coding, prevalence

## Abstract

**Background:**

Widespread lack of awareness and limited real‐world prevalence evidence have impeded cachexia care and research. We hypothesized that healthcare professionals may identify the term cachexia, leading to International Classification of Diseases (ICD) coding for this term, with or without records of body weight loss for diagnosing cancer cachexia, and that frequently, ICD coding does not accurately reflect weight data.

**Methods:**

We assessed cachexia prevalence in patients diagnosed with cancer, using the Clinical Data Warehouse of a French hospital containing two types of real‐world digitized data: (a) ‘structured’: coded diagnoses and electronic records; (b) ‘unstructured’: uncoded clinical narratives/reports: discharge summaries, procedure results, letters. Two sequential searches covering 2018–2023 (1) determined the prevalence of cachexia in all patients with a diagnosis of cancer using ICD‐10 code R64 (cachexia), electronic records of body weight and unstructured narrative data; and (2) examined data of cancers of high‐prevalence cachexia: colorectal, pancreatic and bronchial/lung cancers, determining (a) prevalence of cachexia by these criteria; (b) extent to which a diagnosis of cachexia was supported by weight loss data; and (c) extent to which the diagnosis of cachexia was not made despite objective weight data indicating its presence.

**Results:**

A total of 76 547 of 737 906 patients had cancer of any primary type; 1856 (2.42%) of these had a cachexia diagnosis: 620 identified by ICD code, 1507 by unstructured data, including 271 by both. Of 6946 patients with colorectal, pancreatic or bronchial/lung cancer, 2033 patients (29.3%) were identified with cachexia by structured and/or unstructured data; both approaches were required to discover cachexia cases. From structured data an ICD‐R64 search found only 254 patients described by the term cachexia, of which 127 had weight data supporting the diagnosis in the electronic medical record. An additional 1340 patients with BMI < 20 kg/m^2^ or weight loss > 5% were not coded as cachectic. Unstructured data for the three cancers identified 439 additional cachexic patients.

**Conclusions:**

(1) Standard ICD code‐searching underestimated cachexia prevalence in all patients and those with high‐prevalence‐cachexia cancers; (2) Many cachexia cases were not diagnosed, although data indicated its presence; (3) Many cachexia cases diagnosed by judgement were not supported by data; (4) Increasing provider awareness of cancer cachexia definitions would likely improve cachexia care and research.

## 1 Introduction

Cancer cachexia is defined as a multifactorial syndrome of weight loss characterized by skeletal muscle loss (with or without loss of fat mass) found in the presence of underlying malignant disease [1]. This international DELPHI consensus group provided provisional diagnostic criterion values of > 5% weight loss in the previous 6 months, or 2%–5% weight loss with either a body mass index (BMI) of < 20 kg/m^2^, or reduced muscle mass [1]. The severity of weight loss was subsequently classified based on both BMI and weight loss Grades 0–4 [2]. These criteria have been validated and are included in the American Society of Clinical Oncology (ASCO) guidelines for the management of cancer cachexia [3].

Cachexia also negatively impacts patients' quality of life, chemotherapy tolerance and survival. Unfortunately, patients with cachexia are often not identified. An international survey of health care professionals in cancer care identified limitations in physician awareness of cachexia diagnostic criteria [4]. The diagnosis of cachexia is not made frequently for individual patients or tabulated in patient populations treated in cancer care institutions [5]. The prevalence of cancer cachexia remains to be fully documented. The point prevalence study of DeWys et al. [6] on the prognostic effect of weight loss prior to chemotherapy evaluated 3000 cancer patients enrolled in the Eastern Cooperative Oncology Group (ECOG) clinical trials. This study is cited often as the source of the widely accepted 5% weight loss cutoff for cachexia; however, the prevalence of 5% weight loss is limited to ECOG trial participants in 1980. The Society for Cachexia & Wasting Disorders (SCWD) published cachexia prevalence data in 2010 [7] with subsequent updates in 2014 and 2016 [8, 9, 10]. A 2024 systematic review [11] of cancer cachexia summarized 125 articles comprising 137 960 patients noted an overall prevalence of cachexia in patients with cancer of 33.0% (95% confidence interval [CI]: 32.8, 33.3), but also noted confounding due to the use of disparate criteria and high risk of bias.

The studies cited above were not conducted with the intent of defining cachexia epidemiology. Identifying the proportion of a population that has a specific disease/condition requires representative data sets. There have been attempts to discover the prevalence of cachexia in large data sets such as the UK Biobank [12], a national hospitalization database in Slovenia [13] and a US‐based cancer registry [14]. However, these sources lack body weight data over time, so the investigators' approach was to search for International Classification of Diseases (ICD) codes for cachexia (ICD‐9 799.4; ICD‐10 R64). In the US‐based cancer registry of 8541 cancer patients, cachexia was observed in 2.4% of patients using the cachexia diagnostic code, with a larger number, 14.7%, identified as having weight loss exceeding 5% [14].

Early detection of cancer cachexia is critical to improve patients' outcomes as well as for patient recruitment in clinical trials to test novel therapies. Recently, the SCWD, the leading academic society in the field of cachexia and sarcopenia, initiated a project for quality improvement for cachexia diagnosis. This report is the result of the initial effort of that initiative, attempting to identify points of cancer care that could provide real world data regarding the prevalence of cachexia, as well as the information used for its diagnosis: weight, height and BMI. This observational study was undertaken at a tertiary hospital in France. We hypothesized that healthcare professionals may identify the term cachexia leading to ICD coding for this term, with or without records of body weight loss for diagnosing cancer cachexia and that frequently ICD coding does not accurately reflect the data.

## 2 Methods

We examined data from the Clinical Data Warehouse of the Rouen University Hospital, Rouen, Normandy, France, a tertiary hospital serving the city of Rouen (population ~116 000) and its region (population ~493 000). Most patients with cancer in Rouen receive their primary anti‐cancer therapy at the regional cancer centre and are seen at the Rouen University Hospital for cancer surgery, emergencies, hepatology/gastroenterology/nutrition, lung cancer treatment and other services.

The Clinical Data Warehouse, EDSaN [15, 16, 17], is an in‐house solution developed to query the Rouen University Hospital's data, from about 2 million patients. Publications arising from EDSaN use are listed on the Warehouse website [18].

EDSaN integrates data from the electronic health record (EHR) and various clinical databases: structured data from biology, virology, diagnoses, medical devices, drug administrations, procedures; and unstructured data such as reports or clinical narratives (e.g., discharge summaries, letters, procedure results, prescription letters). The French Commission on Informatics and Liberty (CNIL) approved EDSaN in October 2020. The CNIL is an independent regulatory body whose mission is to ensure that data comply with privacy laws. The CNIL has verified the consistency of EDSaN with General Data Protection Regulations (GPDR) (EU 2016/679). The data are pseudonymized or de‐identified in EDSaN to preserve patient anonymity.

EDSaN consists of a query tool for data search by specific criteria and a selection tool that allows the user to filter and explore datasets collected. EDSaN contains two types of digitized data relevant to this inquiry: ‘structured’ data, consisting of coded diagnoses and a standardized digitized table containing weight, height and BMI, within the EHR; and ‘unstructured’ data, consisting of uncoded information such as narrative clinical notes or reports, including discharge summaries, procedure results and letters. For the unstructured data search, EDSaN combines several Natural Language Processing algorithms used to query narrative data for reference to terms related to cachexia. The algorithms are designed to ensure that searched keywords are relevant (e.g., not in a negative sentence). A specific module was developed to allow semantic expansion to maximize the recall of the query (minimizing the rate of the false negative). This module is based on using all the synonyms from various health terminologies and ontologies, which share the same Concept Unique Identifier of the Unified Medical Language System (UMLS) [19]. Over 750 000 different health concepts are integrated in the module for the French language. This is the largest health dictionary available in French [17]. The structured documents were searched with ICD codes R64 and C*. Unstructured documents were searched using the following terms: (cachexia OR cachectic OR ‘pathological emaciation’ OR sarcopenia) AND (cancer OR ‘malignant tumor’ OR carcinoma OR lymphoma OR leukemia OR melanoma).

**FIGURE 1:**
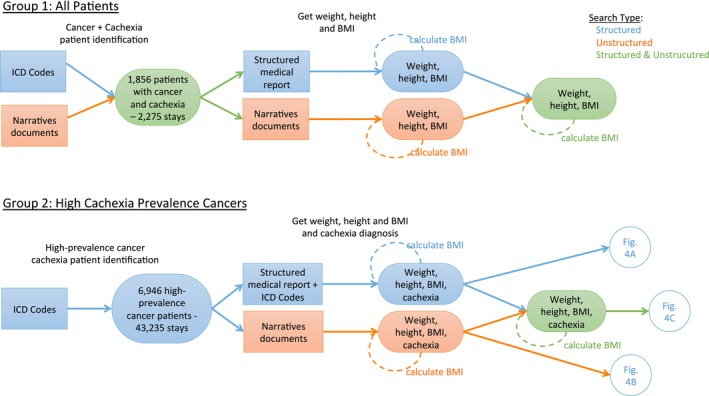
Search design. Two searches conducted included all patients with a diagnosis of cancer (upper panel) and patients with high‐cachexia‐prevalence cancers (lower panel). For all patients of both groups that were found, we extracted from the structured and unstructured data, in addition to the cachexia diagnostic code R64, the patient's height, weight and BMI, if present during the same period. If BMI was missing, it was calculated if the patient's height and weight were available. Structured search indicated by blue, unstructured search indicated by orange, combined structured and unstructured search indicated by green. Data sources are in rectangles; data/results are in ovals. The results of the searches for Group 2 point to the circles indicating the relevant portions of the data display (Venn diagrams) in Figure 4.

We conducted two separate, sequential searches covering the same period, 2018–2023, to gain a recent perspective (Figure 1). We included all out‐patient visits and hospitalizations in this period. We did not perform an a priori power analysis. The first search (Group 1) was designed to determine the prevalence of cachexia in all patients with a diagnosis of cancer identified by ICD 10 codes C*. This file was searched for the ICD 10 code R64 (cachexia). Contemporaneously, we also collected data automatically for BMI, weight and height (and calculated BMI when it was missing, using the latter two when available) using both structured and unstructured records, and where available, these were used to make a diagnosis of cachexia using the diagnostic criterion of > 5% weight loss in the previous 6 months or 2%–5% weight loss with BMI of < 20 kg/m^2^ [1]. A sample of data was assessed to ensure its accuracy.

The second search (Group 2: high‐cachexia‐prevalence cancers) was a subset of patients with at least one of the three cancers considered to have the highest prevalence of cachexia in the literature: malignant colorectal, pancreatic and bronchial/lung tumours. Group 2 patients were identified by ICD 10 codes for the selected primary cancers: C18* or C20* or C21* or C25* or C349, and by indication of any of the three cancers in the narrative documents. The search was designed to determine (a) the prevalence of cachexia in these three cancers as determined by structured and unstructured data; (b) the prevalence of cachexia in these three cancers as determined by the international consensus criteria as above for the all‐cancers cohort [1]; (c) the extent to which clinicians' diagnosis of cachexia was supported by weight data; and (d) the extent to which the diagnosis of cachexia was not made despite the presence of weight data indicating its presence. For patients found by this ICD search, the documents (unstructured data) for each patient were also accessed.

This study was approved by the Rouen University Hospital Scientific and Ethics Committee (CSE2024‐DA065).

## 3 Results

**FIGURE 2:**
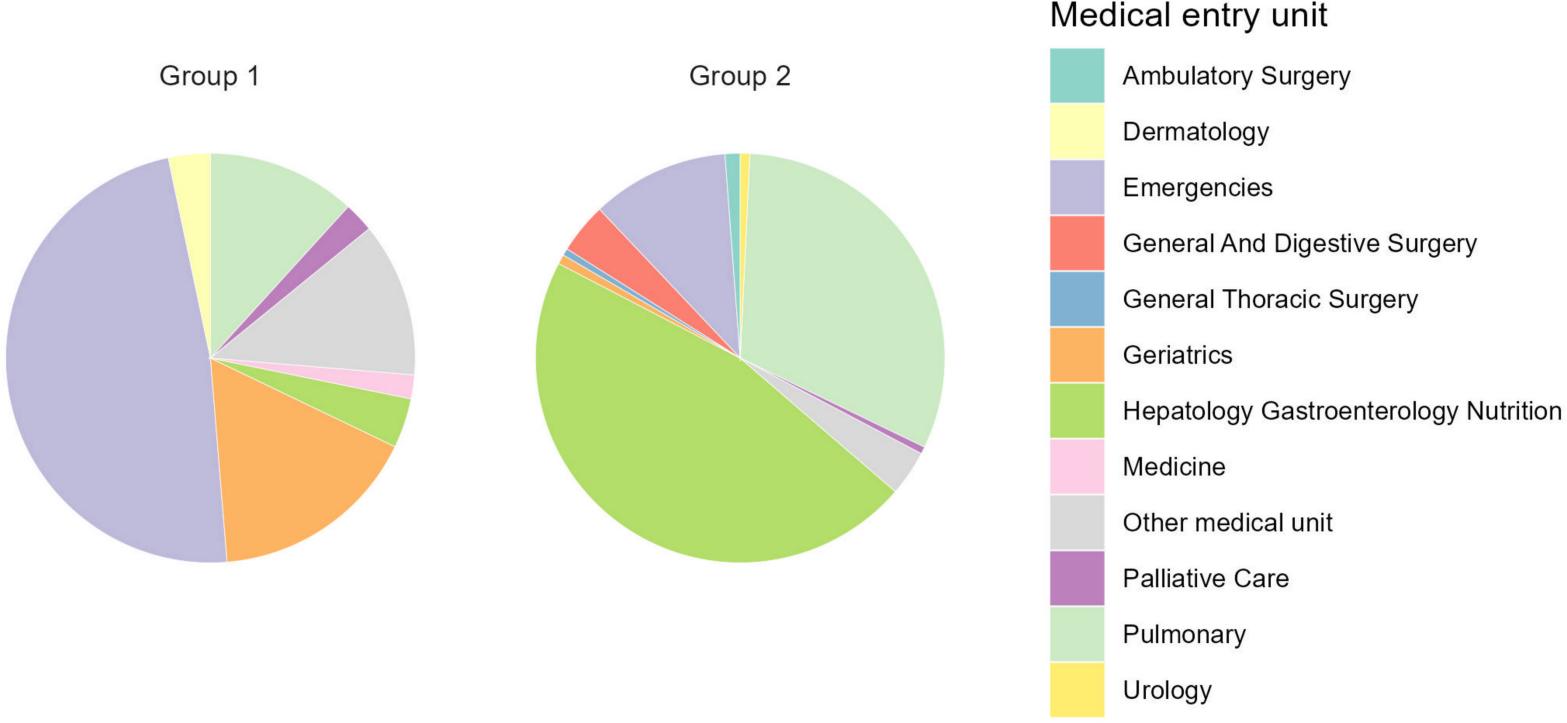
Medical unit of entry to hospital in all patients with cancer (Group 1) and patients with high‐cachexia‐prevalence cancers (Group 2).

The total number of patients in the database for the period searched, 2018–2023, was 737 906. All hospitalizations and out‐patient visits for each patient were included in the search. The initial search revealed 76 547 patients (10.4%) had a diagnosis of cancer of any primary site. The medical unit of entry for each hospital encounter is illustrated in Figure 2. For Group 1, with all types of cancer, the main units were the emergency department and geriatrics (neither service exists at the regional cancer centre), followed by pulmonology and other units.

For Group 2 with high cachexia‐prevalence‐cancers (colorectal, pancreatic and lung), the medical units of entry were the hepatology/gastroenterology/nutrition unit and the pulmonology unit. Surgery and chemotherapy for gastrointestinal cancers are carried out mainly in hospital; treatment of lung cancer is provided in the pulmonology unit.

### 3.1 Analysis of Data From Group 1 (All Cancers)

**FIGURE 3:**
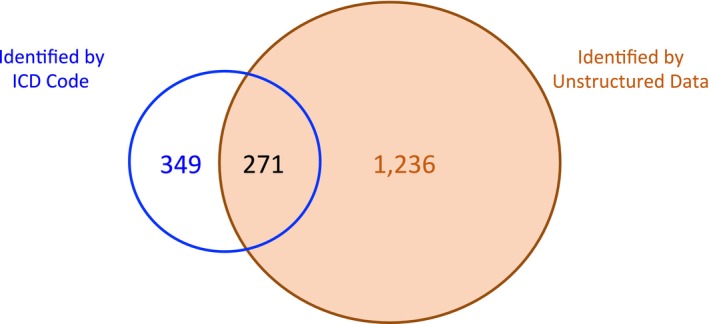
Venn diagram showing patients identified with cachexia among all patients with a diagnosis of cancer (Group 1). A combination of structured and unstructured data found 1856 patients with cachexia, among 76 547 patients with any cancer diagnosis. The salmon‐coloured circle displays those patients identified by searching unstructured data (1507: 1236 + 271); the blue circle displays those patients identified by searching ICD code (620: 349 + 271).

This initial search was conducted as a scoping overview of cachexia in the clinical record of the 76 547 patients that were identified with cancer. Further inquiry into the records of these patients using all available structured and unstructured data revealed a total of 1856 patients that could be classified as having cachexia (2.42% of all patients with cancer). Patients with cachexia had 2275 hospital admissions (median 1.0 [Q1: 1.0—Q3: 1.0]) corresponding to cancer and cachexia criteria. In the records available for these admissions, 620 patients were identified by ICD 10 code R64, of which 349 were not identified otherwise. Unstructured data identified 1507 patients, of which 1236 patients were identified only from ‘unstructured’ data, and 271 were found with both search methods (Figure 3; Table 1). Had the search been conducted using only the ICD code it would have resulted in determining that cachexia was present in 0.81% (620/76 547) of cancer patients, rather than the 2.42% (1856/76 547) found with the broader search.

**TABLE 1:** Group 1 patients. All patients with a diagnosis of cancer.

Patient category	*N* (%)
Database for 2018–2023	737 906
Cancer	76 547 (10.4)^a^
Cancer and cachexia terms	1856 (2.42)^b^
Cachexia found by structured search (ICD 10 R64)	620 (33.4)^c^
Cachexia found by unstructured search	1507 (81.2)^c^
Cachexia found by both structured and unstructured searches	271 (14.6)^c^
**Patients found by both structured and unstructured searches with missing data:**	
Missing either or both BMI/weight loss	648 (34.9)^c^
Missing only BMI data	95 (5.12)^c^
Missing only weight loss data	167 (9.00)^c^
Missing either BMI or weight loss data	262 (14.1)^c^
Missing both BMI and weight loss data	386 (20.8)^c^
**Patients found by structured search with missing data:**	
Missing either or both BMI/weight loss	210 (33.9)^d^
Missing only BMI data	41 (6.61)^d^
Missing only weight loss data	33 (5.32)^d^
Missing either BMI or weight loss data	74 (11.9)^d^
Missing both BMI and weight loss data	136 (21.9)^d^

^a^ Percent of all patients in the database for 2018–2023.

^b^ Percent of patients with cancer.

^c^ Percent of the 1856 patients found by both structured and unstructured searches.

^d^ Percent of the 620 patients found by structured search.

For the 1856 patients found by this search as having cancer cachexia, the complete weight loss and BMI data that would enable one to diagnose the disorder according to the international consensus definition was not present in 648 patients (Table 1). For 386 of these 648 patients, neither weight loss nor BMI was documented or able to be calculated from available data.

### 3.2 Analysis of Data From Group 2: High‐Cachexia‐Prevalence Cancers

This search using ICD 10 codes along found 6946 patients with one or more of colorectal (2924), pancreatic (1202) or non‐small cell lung (2908) cancer. The total of the three individual cancers exceeds the total number of patients found, 6946, as some had more than one primary malignancy. This high‐cachexia‐prevalence group of 6946 patients included a total of 43 235 admissions or out‐patient visits (median 2.0 [Q1: 1.0—Q3: 6.0]) mentioning cancer. Of the 6946 patients, in 2866 (41.2%) the presence of cachexia could not be confirmed owing to missing data for weight loss, BMI or both. Of these, data were missing for both BMI and weight loss for 1577, for BMI or weight loss for 1289, for BMI alone for 249 and for weight loss alone for 1040 patients.

**FIGURE 4:**
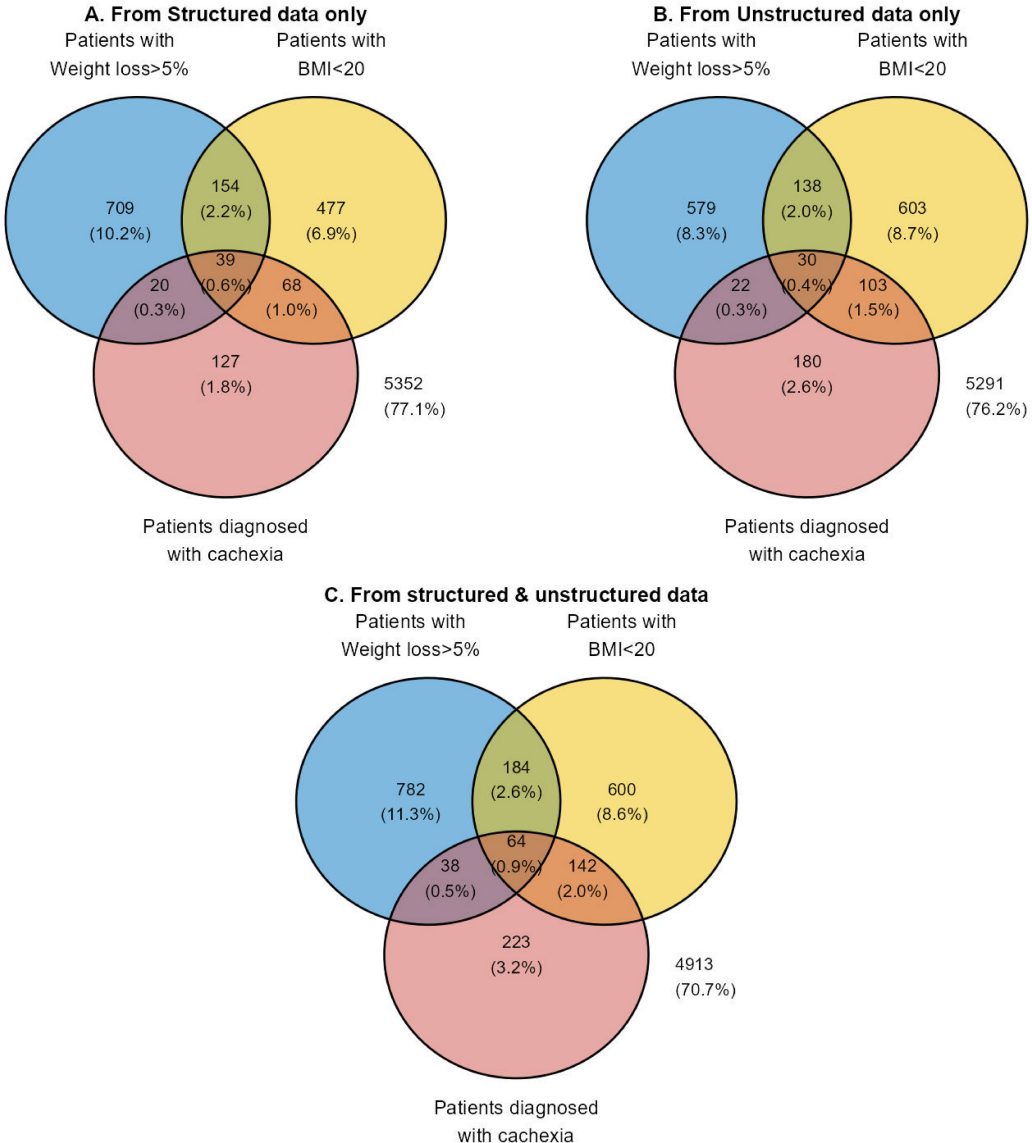
Venn diagrams illustrating data sources for diagnosis of cachexia in high‐cachexia‐prevalence cancers (Group 2). Panel A: Patients identified from structured data sources only; Panel B: Patients identified from unstructured data sources only. Panel C: All data. Blue circles are patients found with weight loss > 5%; yellow circles are patients found with BMI < 20; salmon circles are patients who had been diagnosed with cachexia. Data in panel “C” is not the sum of the data from “A” + “B”, as some patients were identified by both “A” and “B” searches. The numbers outside the circles are those (and %) of the 6946 who are not represented within the circles.

Diagnosis of cachexia from structured data included 1594/6946 (22.9%) of Group 2 patients; they were identified as cachexic by either ICD‐10 R64 or having had data meeting the diagnostic criteria. A Venn diagram (Figure 4A) illustrates the data sources for the diagnosis, of which a small minority (254/1594; 15.9%) were identified by ICD 10 R64 code. Thus, ICD code alone substantially underestimated cachexia prevalence in the structured data. The EHR identified an additional 1340 patients by weight/BMI criteria: weight loss > 5% (863); BMI < 20 kg/m^2^ (631). It is evident from the overlapping zones of the Venn diagram that most patients had just one criterion of weight loss, BMI or ICD code to identify them as affected by cachexia. It is also evident that ICD R64 coding was not substantiated by weight or BMI data in 50% (127/254) of these patients.

Unstructured data search of Group 2 patients (Figure 4B) added a substantial number of patients not found in the structured search (Figure 4A). The Venn diagram for the unstructured search again shows that most patients had data of just one criterion (non‐overlapping portions of the circles) of BMI, weight loss or Natural Language terms for cachexia.

Of the 6946 patients with colorectal, pancreatic or bronchial/lung cancer, a total of 2033 patients (29.3%) were identified as having cachexia supported by structured and/or unstructured data (Figure 4C). Both structured and unstructured searches, as well as weight loss and BMI data, discovered cases of cachexia that the other search method did not. Considering all patients identified (Figure 4C), both by cachexia diagnosis and data for BMI and weight loss, the ICD code R64 and the term ‘cachexia’ identified only 467 patients, of which the code identified only 254 patients, reflecting a minority use of this ICD code. About half of patients identified by ICD code or the term ‘cachexia’ (47.8%; 223/467) did not have BMI or weight loss data supporting the diagnosis. Had the searches been conducted using only the ICD code it would have resulted in determining that cachexia was present in 3.66% (254/6946) of the patients with at least one of the three cancers, rather than the 29.3% (2.033/6946) identified by the broader searches.

## 4 Discussion

A 6‐year review of admissions to a tertiary hospital in Rouen France included 76 547 patients with a diagnosis of cancer. In this setting, the main findings of our study are: (a) searching for cancer cachexia using the standard ICD codes alone does not identify the majority of patients with the disorder, thus greatly underestimates the prevalence when examining all cancers, as well as those known to have a high prevalence of cachexia; (b) many cases of cachexia are not diagnosed, despite the presence of data indicating the presence of cachexia; (c) many cases of cachexia assigned an ICD code by clinicians' observation were not supported by BMI or weight loss data; (d) many patients' cachexia status remains unknown, absent any record of weight history or BMI.

Our study based in an institutional Clinical Data Warehouse has some similarities with other investigations conducted in other biomedical databases. In the UK Biobank, a large‐scale prospective observational population cohort, 5397 of 496 300 (1.087%) biobank participants had cachexia by ICD‐10 codes [12]. In the national database of hospitalizations in Slovenia, 5 484 103 hospitalizations were screened for ICD codes for cachexia, and the prevalence of cachexia was 1.2%–1.9%, being most prominent in cases of cancer and chronic obstructive pulmonary disease [13]. In the only cancer‐specific inquiry [14] of 8541 patients in a state cancer registry, cachexia by ICD 9 code was observed in 2.4% of patients. These numbers for cachexia prevalence are minimal and not at all consistent with those derived from weight measurements on populations of cancer patients [11]. Anker et al. noted that the underreporting of cachexia contributes to its classification as an orphan disease in the United States and the EU [10]. Under‐reporting is not unique to cachexia. Weight‐related disorders are generally underrepresented in ICD codes. Previous research examining the validity of ICD codes to define obesity indicates that these codes substantially underestimate the prevalence of obesity [20, 21]. Underdiagnosis of cachexia has also been highlighted by authors who have summarized prevalences reported in clinical cancer research studies. Takaota et al. demonstrated substantial variability in cachexia prevalence in a systematic review of 125 articles [11] depending on the diagnostic criteria used. Similarly, Anker et al. [10], in reviewing 21 studies reporting cancer cachexia prevalence, also pointed out discrepancies due to varying inclusion criteria and diagnostic approaches. Both authors noted that consistent criteria that reflect the severity of cachexia permitted more reliable prevalence estimates and demonstrated a stronger relationship between cachexia and overall survival [10, 11].

The ground truth, that is, the true prevalence of cancer cachexia can only be assessed by applying diagnostic criteria to each patient's entire weight history. We could find only a single report with extensive longitudinal weight records [22]. These authors studied 10 802 body weight records of 801 patients with gastrointestinal malignancies in the Integrated Data Repository of a single hospital, plotting body weight evolution over average follow‐up times of 230.6 ± 244.4 days. This approach allowed them to characterize the development of deepening weight loss over time. The highest mean weight loss (−48.8% of body weight) was observed in patients with gastric cancer, followed by pancreatic cancer (−35.8%), with the least amount of weight loss reported by patients with colorectal cancer (−12.0%). This type of study can robustly reveal differences between primary cancers. Another key issue is that such data show that overall weight loss greatly exceeds the 2%–5% thresholds that form the initial diagnostic criteria. Cachexia diagnosis should reflect its severity [1, 2] and such data would allow for that.

A key insight from our study is the frequent absence of any weight‐related data in the clinical record of an institution that treated tens of thousands of patients with cancer. The inclusion of BMI and weight loss data is vital not only for clinical care but also for research. Cachexia is increasingly recognized as an area of unmet medical need, and ongoing clinical trials are focusing on novel therapeutic approaches. Without consistent documentation of BMI and weight loss, clinicians may miss early‐stage cachexia, thus delaying intervention and affecting patient recruitment for these trials. Our findings confirm that many healthcare professionals use the term cachexia or synonyms subjectively for diagnosing cancer cachexia rather than the established criteria and emphasize the need for more rigorous documentation to identify cachexic patients and, thus, improve both clinical and research outcomes.

Overall, the diagnosis of cachexia was made in a much higher proportion of patients in the high prevalence group compared to the overall population with cancer, possibly related to their higher intake into hepatology/gastroenterology/nutrition, lung cancer and surgical units, as well as their more numerous admissions to hospital. In these settings, staff would be expected to be more aware of cachexia. Unfortunately, owing to missing data for weight loss, BMI or both for a substantial proportion (41.2%) of patients with high cachexia prevalence cancers, it cannot be confirmed whether they did or did not have cachexia.

ICD code use is clearly minimal for cachexia, but for reasons unknown. Perhaps physicians equate this term with emaciated body habitus (i.e., cachexic) as suggested by survey results [4], which do not ensue early in the progression of cancer‐associated weight loss. Another crucial aspect highlighted in our study is the importance of properly assigning ICD codes for cachexia. Many cases of cachexia were either not diagnosed or underdiagnosed due to a lack of standardized coding practices. Ensuring that each diagnosed case of cachexia is correctly coded is not only essential for accurate prevalence estimates but also for improving patient access to emerging treatments and ensuring that patients can benefit from needed clinical trials and novel therapies.

This study has several limitations. First, the searched database was for a single institution. Behaviour for coding diagnoses differs at other institutions within France and in other countries. Second, the standard digitized form integrated into the medical records of patients at our institution is likely present in most hospitals in France but may not be in other countries. Third, many patients did not have accessible data (BMI, weight loss) that would permit evaluation as to whether they met the criteria for cachexia. However, having these data for additional patients would serve to increase the number of patients established as cachectic, but had not been so diagnosed. As such, this study's principal findings would not have changed, as the difference between those diagnosed with cachexia and those with the disorder would have only increased. Additionally, the validated Grades 0–4 [2] for the severity of cancer‐associated weight loss could not be applied to patients in this sample because both weight loss and BMI are required. Also, at the point of care, body weight data buried in the narrative notes have limited accessibility, and even if they are to hand, the BMI and % weight loss would have to be calculated manually.

Our study supports the growing body of literature stressing the need for more comprehensive use of diagnostic criteria and improved coding practices for cancer cachexia. Addressing these gaps is likely to enhance both the care of patients with or at risk of cachexia, as well as research efforts.

## Funding

This work was supported by the Society on Sarcopenia, Cachexia and Wasting Disorders.

## Conflicts of Interest

The authors declare no conflicts of interest.
